# Mechanistic and therapeutic insights into the function of NLRP3 inflammasome in sterile arthritis

**DOI:** 10.3389/fimmu.2023.1273174

**Published:** 2023-10-25

**Authors:** Yi Xiao, Li Zhang

**Affiliations:** ^1^ Children’s Hospital, Zhejiang University School of Medicine, Hangzhou, China; ^2^ Department of Orthopedics, Hangzhou Medical College, Zhejiang Provincial People’s Hospital, Hangzhou, Zhejiang, China

**Keywords:** NLRP3 inflammasome, pyroptosis, inflammation, arthritis, immune system

## Abstract

The NLRP3 inflammasome, which belongs to the pyrin domain containing 3 family of NOD-like receptors, has a significant impact on both the innate and adaptive immune responses. Regulating host immune function and protecting against microbial invasion and cell damage, the NLRP3 inflammasome plays a crucial role. By triggering caspase-1, it facilitates the development of the inflammatory cytokines IL-1β and IL-18, and triggers cell pyroptosis, resulting in cell lysis and demise. Common sterile arthritis includes osteoarthritis (OA), rheumatoid arthritis (RA) and gouty arthritis (GA), all of which manifest as bone destruction and synovial inflammation in a complex inflammatory state, placing a significant medical burden on the families of patients and government agencies. In the past few years, there has been a growing interest in investigating the impact of cell pyroptosis on arthritis development, particularly the widespread occurrence of pyroptosis mediated by the NLRP3 inflammasome. The NLRP3 inflammasome’s biological properties are briefly described in this review, along with the presentation of the fundamental processes of pyroptosis resulting from its activation. Furthermore, we provide a summary of the advancements made in studying the NLRP3 inflammasome in various forms of arthritis and enumerate the intervention approaches that target the NLRP3-mediated pyroptosis, either directly or indirectly. These discoveries lay the groundwork for future investigations on medications for arthritis, offering fresh approaches for the clinical identification and treatment of this condition.

## 1 Introduction

The immune system defends against internal and external dangers by utilizing innate and adaptive immunity (1). The innate immune response acts as the initial barrier against foreign invaders, functioning as a complex mechanism to detect signals indicating potential threats. Various pattern-recognition receptors (PRRs) are employed by the innate immune system to identify consistent microbial patterns. These PRRs are expressed by a range of cells including macrophages, monocytes, dendritic cells, neutrophils, and epithelial cells (2). The NOD-like receptor (NLR) is a typical type of intracellular PRRs that identifies both pathogen-associated molecular patterns (PAMPs) and danger-associated molecular patterns (DAMPs) derived from the host. It is worth noting that DAMP-induced inflammation, referred to as “sterile inflammation”, occurs in the absence of any external pathogens, which is especially significant in inflammatory diseases. The host strictly controls inflammation, which is a defensive immune response, but excessive inflammation can result in chronic or systemic inflammatory disorders (3).

NLRP3 belonging to NLR family can form NLRP3 inflammasome after PAMPs or DAMPs stimulation which is currently the most fully characterized inflammasome (4). NLRP3 is an intracellular sensor that recognizes various external pathogen irritants and internal danger signals, leading to the assembly and activation of the NLRP3 inflammasome. The primary role of NLRP3 inflammasome is to trigger proteolytic enzyme caspase-1, which leads to the release of the proinflammatory cytokines IL-1β and IL-18, and initiates pyroptosis mediated by gasdermin D (GSDMD) (5). Pyroptosis leads to cellular swelling and dissolution, resulting in the direct release of cytosolic substances and proinflammatory cytokines. This impacts cell function and intensifies the inflammatory response, leading to inflammatory damage and exerting a cytotoxic effect (6). The pathogenesis and development of common sterile arthritis are significantly influenced by the abnormal activation and regulation of the NLRP3 inflammasome. In recent years, there has been a growing interest in the associations between arthritis and NLRP3-mediated pyroptosis (7).

Arthritis commonly presents with symptoms like pain, joint stiffness, and impaired function, all of which are associated with various pathological mechanisms including synovial fibrosis, matrix degradation and chronic inflammation. The widespread occurrence of arthritis impacts various segments of the population, particularly the elderly, leading to a considerable burden on families and communities despite advancements in hospital support (8). Recent studies have indicated that an elevated NLPR3 inflammasome-induced pyroptosis is considered a characteristic feature of the aging phenomenon (9) and occurs in the crucial development of arthritis (10). Hence, it is crucial to investigate the possible mechanisms and efficacious medications for arthritis. Targeting the NLRP3-mediated pyroptosis could hold great promise for anti-inflammatory treatments of arthritis. This review provides an overview of the features and processes involved in the pyroptosis mediated by the NLRP3 inflammasome, emphasizing its crucial contribution to the development of arthritis. Furthermore, we offer an extensive analysis of the potential medications targeting NLRP3-mediated pyroptosis as a therapeutic approach for arthritis. We present encouraging tactics for arthritis treatment and propose future directions for drug development.

## 2 NLRP3 Inflammasome

**Figure 1:**
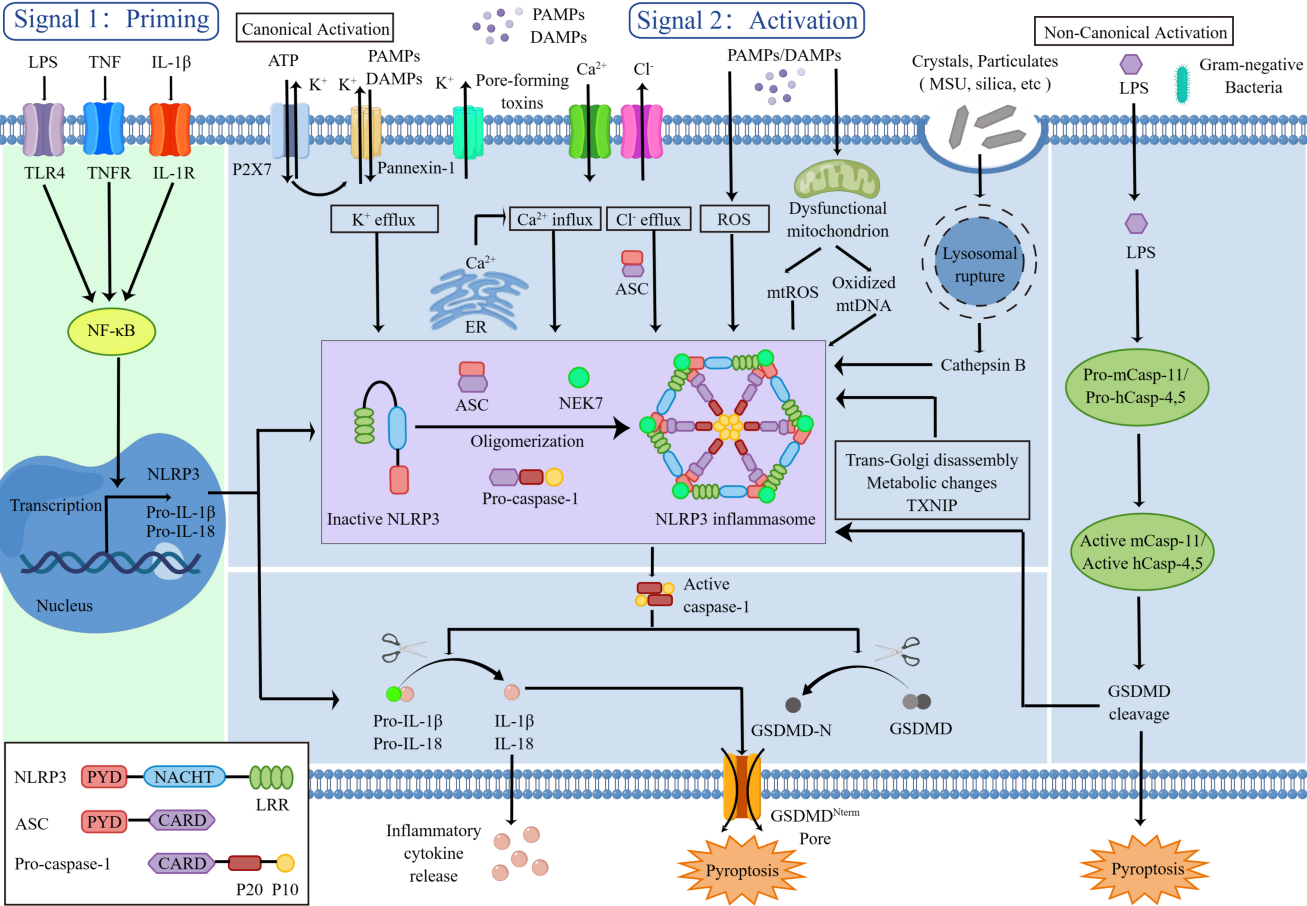
NLRP3 inflammasome priming and activation.

The NLRP3 inflammasome comprises a sensor (NLRP3), an adaptor (ASC) and an effector (caspase-1). NLRP3 is a tripartite protein: (a) one pyrin domain (PYD) in N-terminal which mediates protein interactions for transduction with ASC; (b) one NACHT domain in central part that is crucial for NLRP3 self-association and functions through ATPase activity; (c) one leucine-rich repeat (LRR) domain in C-terminal functions in ligand sensing and autoinhibition by folding back onto the NACHT domain. And the adaptor ASC includes PYD in N-terminal and caspase-recruitment domain (CARD) in C-terminal. In addition, full-length caspase-1 contains CARD in N-terminal, large catalytic domain (p20) in central part and small catalytic subunit domain (p10) in C-terminal (4) (**Figure 1**). Caspase-1 is produced as an inactive zymogen, and its potent cellular functions are tightly regulated by proteolytic activation (2).

NLRP3 is triggered by a diverse range of PAMPs and DAMPs, including crystalline and particulate substances (like monosodium urate (MSU) crystals, silica, asbestos and alum), extracellular adenosine triphosphate (ATP), hyaluronan, pore-forming toxins, RNA-DNA hybrids, as well as numerous fungal, bacterial, viral and protozoan pathogens (11). In the absence of immunological activators, the internal interaction between the NACHT domain and LRR domain hinders the formation of the NLRP3 inflammasome. When stimulated, NLRP3 opens up and oligomerizes by homotypic interactions between NACHT domains. Several researches have indicated that NIMA-related kinase 7 (NEK7) which is a serine-threonine kinase known to be involved in mitosis can bind to the NLRP3 LRR domain to mediate NLRP3 oligomerization (12–14). Then, the homotypic PYD-PYD interaction fosters the recruitment of ASC and the development of nucleates helical ASC filament. Subsequently the ASC CARD domain recruits the CARD domain on procaspase-1, give rise to the NLRP3 inflammasome. Formation of NLRP3 inflammasome complex triggers procaspase-1 self-cleavage, releasing the active capase-1 p20-p10 heterotetramer. On the one hand, the active caspase-1 cleaves immature pro-IL-1β and pro-IL-18 to active forms that are secreted which inducing inflammatory response. On the other hand, the active caspase-1 cleaves GSDMD to initiate pyroptosis which leading to cell death and intensifying the inflammatory response (1, 4).

The NLRP3 inflammasome activation involves two key steps, priming and activation. Priming serves at least two functions as a preparatory stage for subsequent response. The first function is to activate nuclear factor kappa B (NF-κB) through PRRs which in turn upregulates transcription of inflammasome-related components, including inactive NLRP3 and pro-IL-1β and pro-IL-18. A prototypical example of priming event is the binding of lipopolysaccharide (LPS) to Toll-like receptor 4 (TLR4) (15). The induction of NLRP3’s posttranslational modifications (PTMs) including ubiquitylation and phosphorylation, which keep it in an auto-suppressed inactive yet signal-competent state, is the second function of priming (16).

The second activation step takes place following the detection of an NLRP3 activator, leading to completely NLRP3 activation and NLRP3 inflammasome assembly (17). The chemical properties and molecular structures of PAMPs and DAMPs stimuli differ, and the direct binding of stimuli to NLRP3 is seldom observed. It’s likely that NLRP3 responds to a common cellular event, but the mechanisms leading to NLRP3 inflammasome activation are intensely debated. Many different mechanisms have been proposed such as efflux of potassium ions (K^+^) or chloride ions (Cl^−^), influx of calcium ions (Ca^2+^), lysosomal disruption, mitochondrial dysfunction and reactive oxygen species (ROS) production, most of which are not mutually exclusive.

(1) Model of Ion Fluxes

The ion fluxes (K^+^ efflux, Ca^2+^ influx, and Cl^-^ efflux) are common triggers of NLRP3 inflammasome activation, of which K^+^ efflux is an indispensable upstream event. Extracellular ATP stimulates P2X purinoceptor 7 (P2X7) -dependent pore formation by the pannexin-1 hemichannel, triggering K^+^ efflux and allowing extracellular NLRP3 agonists to enter the cytosol and directly engage NLRP3 (18, 19).

It has also been suggested that Ca^2+^ mobilization is a vital upstream event in NLRP3 activation. Ca^2+^ is mobilized by either opening the plasma membrane channels or releasing the endoplasmic reticulum (ER)-related intracellular Ca2+ stores to allow the influx of Ca^2+^ to the cytosol (20, 21). In addition, chloride intracellular channels (CLICs)-dependent Cl^-^ efflux is an essential and proximal upstream event for NLRP3 inflammasome activation (22, 23).

(2) Model of Lysosomal Disruption

Crystalline or particulate NLRP3 agonists such as MSU, silica and alum are engulfed, and their physical characteristics lead to lysosomal rupture, resulting in cytosolic release of lysosomal contents that are somehow sensed by the NLRP3 inflammasome. The lysosomal protease, cathepsin B, is a vital triggered signal to NLRP3 inflammasome (24).

(3) Model of Mitochondrial dysfunction and ROS

All DAMPs and PAMPs, including ATP and particulate/crystalline activators, trigger the generation of ROS. The mitochondria are the main intracellular organelles that contribute the most to cellular ROS to activate NLRP3 inflammasome complex formation (25). Mitochondrial dysfunction also releases mtDNA into the cytosol and the mtDNA subsequently oxidized after stimulation. The oxidized mtDNA specifically activated the NLRP3 inflammasome (26).

Other factors can also activate the NLRP3 inflammasome such as trans-Golgi disassembly, metabolic changes, and the thioredoxin-interacting protein (TXNIP) activity.

Besides the canonical mode of inflammasome activation, other caspases (caspase-11 in murine, caspase 4/5 in humans), also play a role in controlling the release of IL-1β/IL-18 and pyroptosis through the non-canonical NLRP3 inflammasome activation. The LPS derived from Gram-negative bacteria can enter the cytosol independent of TLR4 and then bind directly to caspase-11 to produce active caspase-11, resulting in GSDMD cleavage and NLRP3 inflammasome activation (5).

IL-1β and IL-18, the most important products of NLRP3 inflammasome activation, belonging to the IL-1 family, are vital for both innate immunity and adaptive immunity. Emerging studies have reported the NLRP3/IL-1 axis plays a role in inflammation and target organ damage, induces naïve T cells differentiation, and amplifies T and B cell responses (27). GSDMD, as another important product of NLRP3 inflammasome activation, serves as the executioner of pyroptosis. The structure of GSDMD consists of three sections from N-terminal to C-terminal, active domain (GSDMD-N), central short linker region and autoinhibition domain (GSDMD-C). Active caspase-1 cleaves GSDMD, removing GSDMD-C and releasing it from intramolecular inhibition (16). Afterwards, the active GSDMD-N then generates membrane pores and leads to IL-1β and IL-18 release, water influx, ion flux, cell swelling, and eventual lysis. Together, the host must tightly control NLRP3 to prevent excessive activation of NLRP3 inflammation, which can lead to chronic or systemic inflammatory conditions like arthritis.

The NLRP3 inflammasome activation involves two key steps, priming and activation. The signal 1 (priming, left) is mediated by cytokines or PAMPs to its receptors, such as LPS binding to TLR4, which activates the NF-κB signaling pathway. NF-κB translocates to the nucleus and binds to the DNA, then promotes the transcription of NLRP3, pro-IL-1β and pro-IL-18, which remain in inactive forms in the cytoplasm. Signal 2 (activation, right) requires subsequent PAMP or DAMP stimulation, such as ATP, pore-forming toxins, crystals and particulates, that activate multiple upstream signaling events. These include K^+^ efflux, Ca^2+^ influx, Cl^-^ efflux, lysosomal disruption, mitochondrial dysfunction and ROS production. Other factors also implicated in the assembly and activation of the NLRP3 inflammasome, including trans-Golgi disassembly, metabolic changes and TXNIP. Once activated, NEK7 promotes oligomerization of NLRP3, ASC and pro-caspase-1 to format NLRP3 inflammasome complex. This complex, in turn, catalyzes the conversion of pro-caspase-1 to active caspase-1, which cleaves the inflammatory cytokines IL-1β and IL-18 to amplify the inflammatory response and cleaves the GSDMD to inducing pyroptosis. In addition, the LPS from Gram-negative bacterial gains can access to the cytosol and then bind directly to caspases (caspase-11 in murine, caspase 4/5 in humans) to produce active caspase, resulting in GSDMD cleavage and NLRP3 inflammasome activation.

LPS, lipopolysaccharide; TLR4, Toll-like receptor 4; TNF, tumor necrosis factor; TNFR, tumor necrosis factor receptor; IL-1β, interleukin-1β; IL-18, interleukin-18; NF-κB, nuclear factor kappa B; NLRP3, the NOD-like receptor family, pyrin domain containing 3; PYD, pyrin domain; LRR, leucine-rich repeat; CARD, caspase-recruitment domain; P20, large catalytic domain; P10, small catalytic domain; ATP, adenosine triphosphate; P2X7, P2X purinoceptor 7; PAMP, pathogen-associated molecular pattern; DAMP, danger-associated molecular pattern; K^+^, potassium; Ca^2+^, calcium; Cl^-^, chloride; NEK7, NIMA-related kinase 7; ROS, reactive oxygen species; MSU, monosodium urate; TXNIP, thioredoxin-interacting protein; GSDMD, gasdermin D.

## 3 The involvement of the NLRP3 inflammasome in arthritis

### 3.1 Osteoarthritis

Osteoarthritis (OA) is a clinically common degenerative joint disorder characterized by synovial hyperplasia, progressive destruction of articular cartilage and secondary osteophytes, with the knee being the main peripheral joint involved (28). The common symptoms of OA are pain and joint stiffness, with a small number of patients eventually experiencing loss of joint function. On a macroscopic level, the causes of OA include a variety of factors such as ageing, obesity, trauma, and excessive joint loading. On a microscopic level, the pathological cause of OA is the imbalance of cellular metabolism in the joint leading to the overexpression of inflammatory factors and alterations in the cartilage matrix microenvironment (29–31). The majority of OA therapeutic treatments currently available are palliative, concentrating on pain management and anti-inflammation. At present, knee arthroplasty is the ultimate treatment option for OA, but it has site restrictions, serious side effects, and is irreversible (32). Understanding the pathological process of OA and further researching effective treatments are vital in order to limit the generation of inflammatory mediators and cartilage breakdown in the joints.

The molecular mechanisms of NLRP3-mediated pyroptosis have received widespread attention during the progression of OA (32), which can lead to the deterioration of cartilage within the joint and inflammation of the synovium. Research indicates higher levels of NLRP3 protein manifestation in synovial specimens obtained from individuals suffering from knee osteoarthritis. The NLRP3 inflammasome speeds up the maturation of IL-1β and IL-18, which have a crucial role in the pathogenesis and progression of OA. Xin et al. (33) confirmed that NLRP3-mediated pyroptosis is closely associated with synovitis in temporomandibular joint osteoarthritis (TMJOA). Additionally, the utilization of the Caspase-1 inhibitor Ac-YVAD-cmk and the NLRP3 inhibitor MCC950 can disrupt the activation of NLRP3 and hinder pyroptosis in synovial cells, ultimately leading to a decrease in local synovitis. Hence, the NLRP3/Caspase-1 signaling pathway plays a crucial role in the initiation and advancement of osteoarthritis, making the pyroptosis process highly significant in this context.

NF-κB is a crucial upstream activator of the NLRP3 inflammasome, which by increasing the expression of NLRP3 related components causes the inflammasome to prime and assemble. Consequently, it may trigger the oversecretion of inflammatory cytokines, accelerating cartilage loss and fibrosis in the knee joint. The purinoceptor P2X7R, which serves as a significant trigger for inflammation, has gained considerable interest as a possible target for treating OA. Li et al. (34) discovered that activated P2X7 receptor enhances extracellular matrix breakdown and induces inflammatory cell pyroptosis in OA chondrocytes via the interaction between NF-κB and NLRP3, thereby worsening symptoms of osteoarthritis. Yan et al. (35) found that Licochalcone A (Lico A) has the ability to hinder the activation of NLRP3 inflammasome and chondrocytes pyroptosis induced by LPS through the Nrf2/HO-1/NF-κB axis, thereby reducing the advancement of osteoarthritis in destabilization of the medial meniscus (DMM)-induced OA mouse model. Additionally, Jin et al. (36) compared the effects of diets rich in different types of dietary fatty acid (FA) on obesity-associated OA and found that diets rich in n-3 PUFAs had anti-inflammatory and anti-pyroptotic properties, the mechanism of which may be closely related to the NF-κB/NLRP3 crosstalk in chondrocytes. Collectively, inhibition of NF-kB signaling to modulate NLRP3-mediated pyroptosis is a potential strategy for OA treatment which could inhibit chondrocyte pyroptosis and increase collagen II expression, thereby attenuating OA progression.

**Figure 2:**
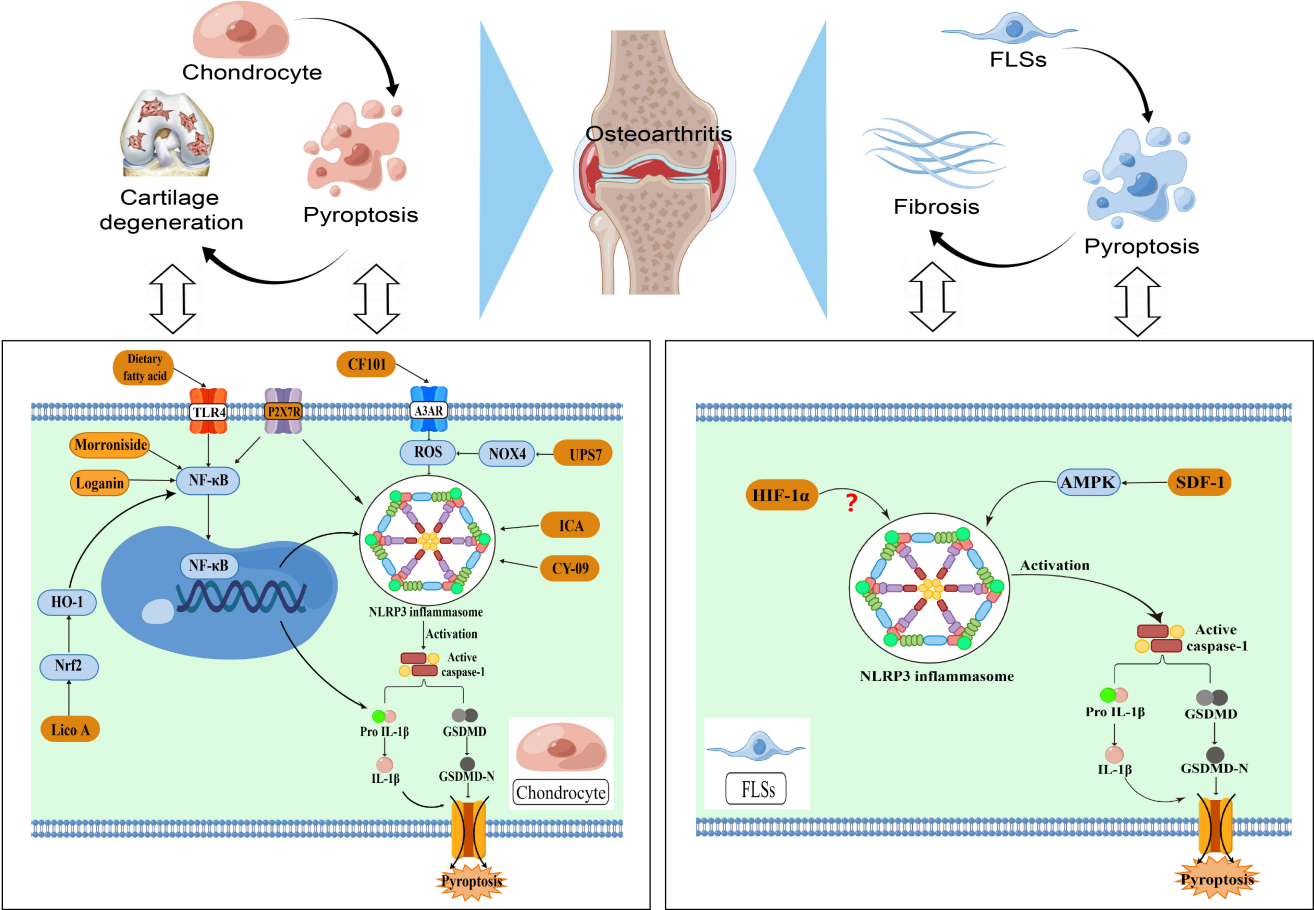
NLRP3-mediated pyroptosis in OA pathogenesis.

Elevated levels of ROS are widely believed to play an essential role in the functional degeneration of articular cartilage during the progression of OA. Hypoxia of synovial tissue is one of the key pathological features of synovitis, and the hypoxic state of the microenvironment within the knee cavity can exacerbate synovial inflammation in a number of ways (31). A series of reactions caused by hypoxia are involved in the pathological processes related to osteoarthritis, including synovial inflammation, cartilage destruction and angiogenesis. Liu et al. (37) demonstrated that ubiquitin specific protease 7(UPS7) promotes OA progression through the NOX4/ROS/NLRP3 axis. The inhibition of NOX4 ubiquitination by UPS7 prevents its degradation, resulting in elevated production of ROS. These ROS can then activate the NLRP3 inflammasome, leading to enhanced secretion of IL-1β and IL-18, as well as the pyroptosis of chondrocytes. The activation of the specific adenosine A3 receptor (A3AR) has demonstrated strong anti-inflammatory effects in numerous diseases. Bai et al. (31) found that activation of A3AR could reduce the severity of anterior cruciate ligament transection (ACLT) model by inhibiting NLRP3-mediated pyroptosis and downstream inflammatory cascades. In particular, the activation of A3AR inhibits the progression of OA and alleviates the perception of pain by blocking the signaling pathway of ROS/NLRP3/GSDMD. Hence, reducing the amount of ROS to weaken the activation of the NLRP3 inflammasome could be a promising approach to intervene in OA and should be explored further (**Figure 2**).

Osteoarthritis involves the degeneration of cartilage, fibrosis of the synovial tissue, and the presence of chronic inflammation. Chondrocytes and fibroblast like synoviocytes (FLSs) play a significant role in the molecular process of NLRP3-mediated pyroptosis in osteoarthritis. The NF-κB signaling and NLRP3 interaction can result in chondrocyte pyroptosis, which in turn enhances the release of inflammatory cytokines and ultimately leads to cartilage degeneration.Additionally, ROS levels play a key role in the degeneration of articular cartilage function. Excessive ROS can activate the NLRP3 inflammasome and its downstream pathways, further exacerbating cartilage loss. The development of synovial fibrosis is primarily caused by the pyroptosis of FLSs, with the NLRP3 inflammasome playing a role in the classical pyroptotic pathway, which worsens the progression of synovial fibrosis. The disparity in cellular metabolism levels within the knee joint causes an overabundance of inflammatory substances and changes in the microenvironment of the cartilage matrix, ultimately leading to the onset of OA.

### 3.2 Rheumatoid arthritis

Rheumatoid arthritis (RA) is a chronic inflammatory bone disease, an autoimmune degenerative disease characterized by joint synovitis, which is characterized by polyarticular and symmetrical damage to the facet joints of the hands and feet, and can eventually lead to joint pain and deformity (38). The NLRP3 inflammasome is closely related to RA, and its abnormal activation is important in guiding the inflammatory response and the development of immune diseases. The NLRP3-mediated pyroptosis could secrete large amounts of inflammatory factors, which penetrate into synovial tissue and lead to synovial hyperplasia, thereby exacerbating the histopathological changes of RA. Wu et al. (39) successfully constructed an adjuvant arthritis (AA) rat model and investigated the role of acid-sensitive ion channels (ASICs) in chondrocyte pyroptosis. The discovery was made that the buildup of inflammatory substances in joints causes a reduction in the acidity of synovial fluid and triggers ASIC1a, which facilitates the entry of calcium ions into cells. Simultaneously, the increase in intercellular calcium promotes the production, clustering, and formation of the NLRP3 inflammasome, subsequently leading to the activation of caspase-1. In addition to showcasing the significance of synovial fluid pH in the development of RA, this study uncovers the role of NLRP3-mediated pyroptosis in the progression of RA.

The activation of the NLRP3 inflammasome is widely recognized as a process that occurs in two steps. As mentioned above, the NF-κB pathway is involved in the initiation of NLRP3 and IL-1β gene transcription in the priming step; and the NLRP3 inflammasome can be activated by PAMPs or DAMPs in the activation step. Various molecules or cellular events, such as malfunctioning mitochondria and mitochondrial DNA, ROS, ion flow, and lysosomal harm, can trigger the activation of NLRP3 (40). Among them, Ge et al. (41) found that punicalagin (PUN), an active substance extracted from pomegranate peel, has the ability to hinder the activation of the NF-κB signaling pathway, consequently restraining the occurrence of pyroptosis in macrophages. Partially alleviating the progression of RA can be achieved by inhibiting the pro-inflammatory impact of macrophages via the NF-κB signaling pathway, offering a fresh perspective for treatment. Hong et al. (42) confirmed that the presence of low oxygen levels in the surrounding area of the joint worsens the development of inflammation within the joint in individuals with rheumatoid arthritis. They discovered that heightened levels of HIF-1α in FLSs could worsen synovitis through FLSs pyroptosis, whereas the rise in GRK2 expression resulting from oxidative stress can contribute to the elevation of HIF-1α expression. Overall, their study showed that hypoxia induced FLSs pyroptosis by activating the ROS/GRK2/HIF-1α/NLRP3 pathway, whereas monomeric derivatives of paeoniflorin (MDP) reduced hypoxia-induced pyroptosis in FLSs by inhibiting the GRK2/HIF-1α axis.

**Figure 3:**
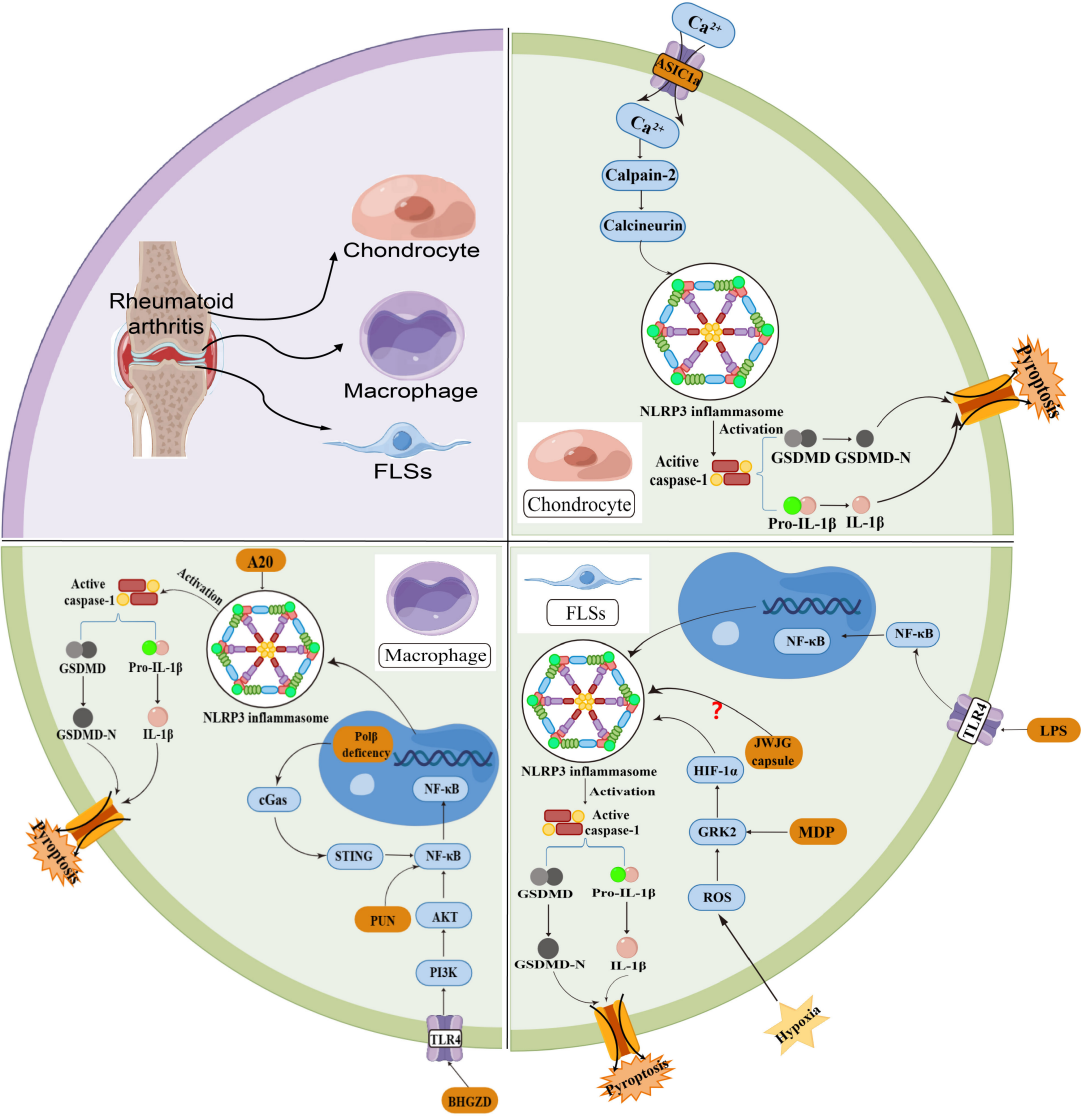
NLRP3-mediated pyroptosis in RA pathogenesis.

The primary mediator of cartilage destruction in rheumatic illnesses is the inflammatory cytokine IL-1, which is mainly derived from macrophages. Walle et al. (43) discovered that the absence of the RA-susceptibility gene A20 in macrophages greatly increased NLRP3-mediated caspase-1 activation, pyroptosis and IL-1β secretion. The findings demonstrate that A20 functions as a new inhibitor of NLRP3 inflammasome activation. MicroRNAs (miRNAs) can regulate cellular processes (cell proliferation, apoptosis, etc.) and are widely involved in inflammatory and pyroptotic responses. Jiang et al. (44) revealed that miR-144-3p could regulate NLRP3-mediated chondrocyte pyroptosis by inhibiting the expression of the target gene PTEN and suppressing the activation of PINK1/Parkin signaling. Further research into the pathogenesis of RA could help develop effective targeted drugs and inhibitors, offering new therapeutic options for the relief of RA symptoms (**Figure 3**).

Multiple immune cells are involved in the development of RA. The basic pathological features of RA include persistent synovial inflammation and articular cartilage degeneration. A decrease in the PH of synovial fluid is one of the physiological manifestations of RA, and extracellular acidosis induces chondrocyte ASIC1a activation, leading to the influx of Ca2+ into cells and activation of NLRP3 inflamasome. The mechanism may be related to the activation of the calpain-2/calcineurin pathway. Macrophage pyroptosis can result in an overactive immune response and the release of inflammatory mediators, which can contribute to the breakdown of bone and cartilage. The NF-κB pathway plays an important interactive role in NLRP3-mediated pyroptosis of macrophage. In FLSs, the classical pyroptotic pathway is mainly activated by ROS and LPS. Overall, multiple cellular pyroptotic pathways mediated by the NLRP3 inflammasome collectively exacerbate the histopathological changes in RA.

### 3.3 Gouty arthritis

Gouty arthritis (GA) is a metabolic disorder resulting from an abnormality in the metabolism of purine. It is characterised by deposits of MSU crystals in the articular cartilage, synovial sac and other tissues stimulating the production of inflammation, which leads to joint pain and swelling (45). MSU deposition can induce the inflammasome and activate the secretion of the inflammatory cytokines IL-1β and IL-18. Meanwhile, studies have reported increased levels of NLRP3, IL-1β and IL-18 in the serum and joint fluid of gout patients compared to healthy controls (46, 47). The NLRP3-mediated pyroptosis may be involved in the crosstalk between MSU-activated gout and the inflammatory cascade. Tian et al. (46) constructed a MSU-induced GA mouse model and found that inhibition of the NLRP3-mediated pyroptosis using inhibitors improved joint inflammation in the mouse model. Targeting the NLRP3 inflammasome could be an efficient approach for managing and preventing GA, as it plays a crucial role in the development and advancement of inflammation and chronic ailments.

Luo et al. (40) found that corilagin has the ability to restrict the generation of mitochondrial ROS, thereby inhibiting the association between TXNIP and NLRP3. In combination, corilagin inhibits the ROS/TXNIP/NLRP3 pathway to suppress inflammasome activation and pyroptosis, indicating its potential as an antioxidant in reducing GA dependent on NLRP3. And Hao et al. (48) proved the important value of the NF-κB pathway in GA development. They used JQ-1, a BRD4-specific inhibitor, to inhibit MSU-induced macrophage pyroptosis and reduce levels of NLRP3 inflammasome. The results indicate that the expression of Brd4 in the joint was associated with the damage to the joint and the occurrence of pyroptosis cascade events in GA through the signaling pathway of NF-κB/NLRP3/GSDMD. Moreover, the P2Y14R, which is a G-protein-coupled receptor, has the ability to suppress the production of cyclic adenosine monophosphate (cAMP) in immune cells, making it a promising candidate for controlling inflammatory reactions. Li et al. (49) found that P2Y14R knockout disrupted MSU-induced histopathologic changes in rat synovium and simultaneously inhibited NLRP3 inflammasome activation in synovial tissues, which was reversed by reducing cAMP levels. The results of their study revealed the involvement of the P2Y14R/cAMP/NLRP3 signaling pathway in acute gouty arthritis, indicating that intracellular cAMP has a distinct function in facilitating communication between P2Y14R activation and the inflammatory process during GA flares.

**Figure 4:**
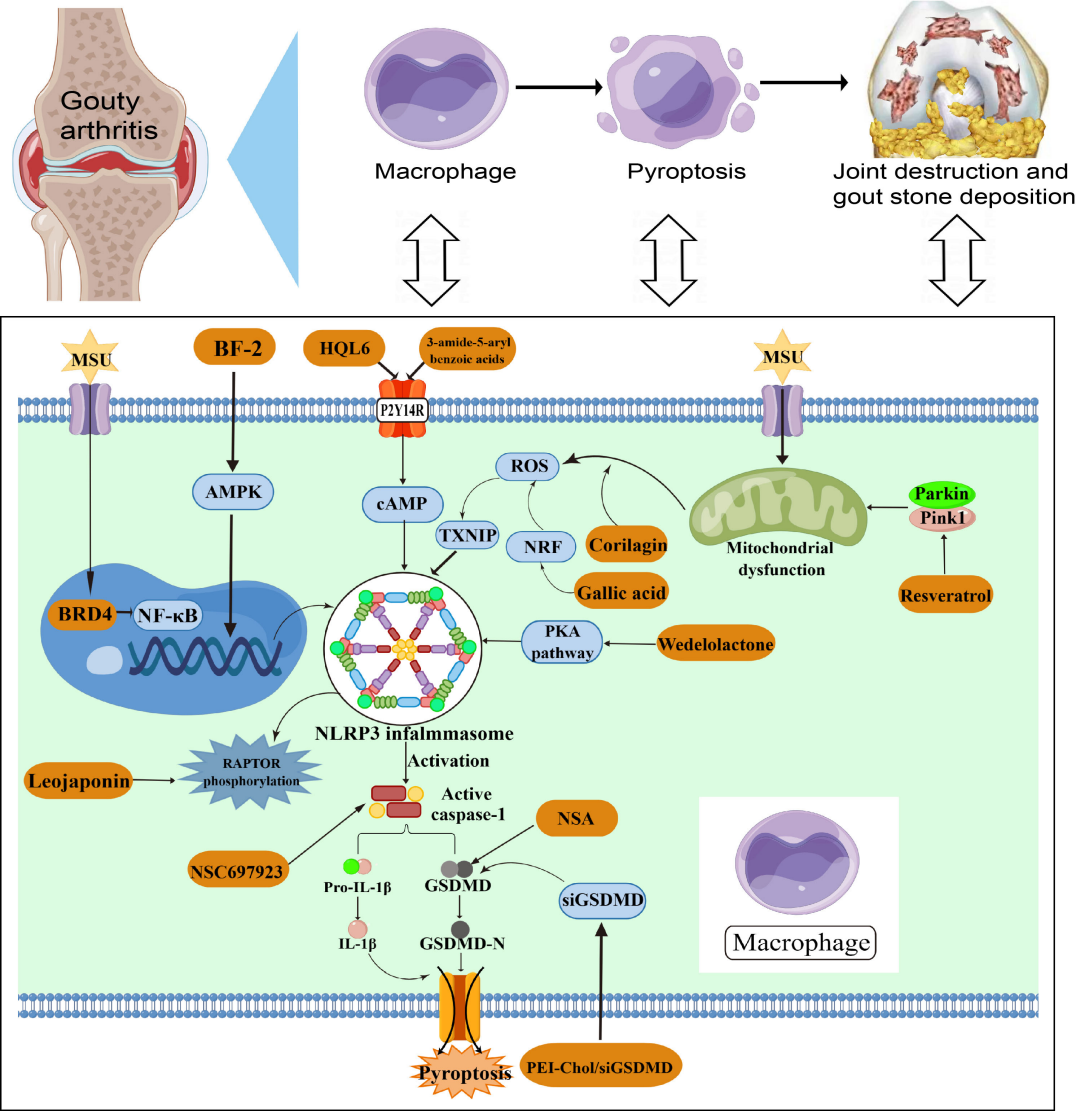
NLRP3-mediated pyroptosis in GA pathogenesis.

Overall, the pathogenesis of GA is relatively complex. The presence of MSU crystals stimulates the generation of diverse inflammatory substances and chemokines, thereby facilitating the infiltration of neutrophils and monocytes (50). The occurrence of GA is significantly influenced by pyroptosis mediated by the NLRP3 inflammasome, and numerous investigations have shed light on the activation or control mechanism of the NLRP3 inflammasome in the progression of GA. However, the exact role and mechanism of NLRP3 inflammasome in GA is still not fully elucidated, and more studies would be necessary to clarity its mechanism with GA in future (**Figure 4**). And the regulatory element of NLRP3-mediated pyroptosis in arthritis are listed in **Table 1**.

**Table 1:** Regulatory element of NLRP3-mediated pyroptosis in arthritis.

Diseases	Regulatory element	Possible upstream or downstream pathways	Target cells	Animal models	Ref
OA	UPS7	NOX4/ROS/NLRP3	Chondrocyte	Rat	(37)
	P2X7	NF-κB/NLRP3/Caspase-1	Chondrocyte	Rat	(34)
	Morroniside	NF-κB/NLRP3/Caspase-1	Chondrocyte	Mice	(32)
	Loganin	NF-κB/NLRP3/Caspase-1	Chondrocyte	Mice	(51)
	ICA	NLRP3/Caspase-1	Chondrocyte	Rat	(52)
	Dietary fatty acid	TLR4/NF-κB/NLRP3/Caspase-1	Chondrocyte	Mice	(36)
	CY-09	NLRP3/Caspase-1	Chondrocyte	Mouse	(53)
	CF101	ROS/NLRP3/Caspase-1	Chondrocyte	Rat	(31)
	Lico A	Nrf2/HO-1/NF-κB/NLRP3	Chondrocyte	Mice	(35)
	HIF-1α	NLRP3/Caspase-1	FLSs	Rat	(54)
	SDF-1	AMPK/NLRP3/Caspase-1	FLSs	Rat	(55)
	MCC950	NLRP3/Caspase-1	Macrophage	Rat	(33)
RA	HQH	NLRP3/Caspase-1	/	Rat	(56)
	ASIC1a	NLRP3/Caspase-1	Chondrocyte	Rat	(39)
	ASIC1a	calpain-2/calcineurin/NLRP3/Caspase-1	Chondrocyte	/	(57)
	Punicalagin	NF-κB/NLRP3/Caspase-1	Macrophage	Mice	(41)
	A20	NLRP3/Caspase-1	Macrophage	Mice	(43)
	Pol β	cGas/STING/NF-κB/NLRP3	Macrophage	Mice	(58)
	BHGZD	TLR4/PI3K/AKT/NF-κB/NLRP3	Macrophage	Rat	(38)
	MDP	ROS/GRK2/HIF-1α/NLRP3	FLSs	Rat	(42)
	LPS	NF-κB/NLRP3/caspase-1/GSDMD	FLSs	/	(59)
	JWJG Capsule	NLRP3/caspase-1	FLSs	/	(60)
	C1q and PTX3	NLRP3/caspase-1	Monocyte	/	(61)
GA	Wedelolactone	PKA pathway/NLRP3	Macrophage	Mice	(62)
	NSA	NLRP3/Caspase-1/GSDMD	Macrophage	Mice	(63)
	PEI-Chol/siGSDMD	NLRP3/Caspase-1/GSDMD	Macrophage	Mice	(64)
	Resveratrol	(Pink1/Parkin pathway)/NLRP3	Macrophage	Mice	(65)
	Leojaponin	RAPTOR/NLRP3	Macrophage	Mouse	(66)
	HQL6	P2Y14R/cAMP/NLRP3	/	Rat	(67)
	Gallic Acid	Nrf/ROS/NLRP3	Macrophage	Mice	(50)
	3-amide-5-aryl benzoic acid	P2Y14R/NLRP3	Macrophage	Mice	(68)
	Corilagin	ROS/TXNIP/NLRP3	Macrophage	Mice	(40)
	BF-2	MAPK/NF-κB/NLRP3	Macrophage	Mice	(69)
	NSC697923	NLRP3/Caspase-1	Macrophage	Mice	(45)
	Brd4	NF-κB/NLRP3/GSDMD	Macrophage	Rat	(48)

ICA, Icariin; HQH, Huai Qi Huang; BHGZD, Baihu-Guizhi decoction; MDP, monomeric derivatives of paeoniflorin; JWJG capsule, Jinwujiangu capsule; NSA, necrosulfonamide; PEI-Chol/siGSDMD, siGSDMD-loaded PEI-Chol lipopolymers; BF-2, Baeckein E.

GA is characterized by MSU deposition stimulating the onset of joint inflammation. The NLRP3 inflammasome is induced by aggregation of MSU during pathogenesis, which then induces the activation of caspase-1 and IL-1β. Multiple pathways including mitochondrial dysfunction, ROS and NF-κB are involved in NLRP3-mediated macrophage pyroptosis in the development of GA. NLRP3 inflammasome activation may be a new target for the treatment of GA.

## 4 Strategies for targeting the NLRP3 inflammasome in arthritis

At this stage, a range of NLRP3 inflammasome-related drugs and intervention strategies for the treatment of arthritis are mainly based on the NLRP3-mediated pyroptosis pathway, and can be divided into the following categories: 1) drugs and tactics that directly block or inhibit the NLRP3-mediated pyroptosis; 2) drugs and tactics that indirectly influence the NLRP3-mediated pyroptosis by modulating its classical activation pathway. Depending on the type of potential treatment, a step-by-step account is given according to the following classification (**Table 2**).

**Table 2:** Drugs targeting NLRP3 inflammasome mediated pyroptosis in arthritis.

Categories	Drug names	Related arthritis	Ref
NLRP3 inflammasome direct inhibitors			
NLRP3 direct inhibitors	ICA	OA	(52)
	CY-09	OA	(53)
	MCC950	OA	(33)
	Leojaponin	GA	(66)
Caspase direct inhibitors	Ac-YVAD-cmk	OA	(29)
	NSC697923	GA	(45)
NLRP3 inflammasome indirect inhibitors			
NF- κB inhibitors			
	Punicalagin	RA	(41)
	Morroniside	OA	(32)
	loganin	OA	(51)
	Dietary fatty acid	OA	(36)
	Lico A	OA	(35)
	BF-2	GA	(69)
ROS or mitochondrial function inhibitors			
	MDP	RA	(42)
	CF101	OA	(31)
	Gallic Acid	GA	(50)
	Corilagin	GA	(40)
	Resveratrol	GA	(65)
ASIC1a inhibitors			
	amiloride	RA	(39)
	PcTx1	RA	(57)
P2Y14 receptor inhibitors			
	HQL6	GA	(67)
	3-amide-5-aryl benzoic acids	GA	(68)
GSDMD inhibitors			
	NSA	GA	(63)
PKA activators			
	Wedelolactone	GA	(62)
AMPK activators			
	SDF-1	OA	(55)
Traditional Chinese medicine			
	HQH	RA	(56)
	BHGZD	RA	(70)
	JWJG capsule	RA	(60)

ICA, Icariin; NSA, necrosulfonamide; BF-2, Baeckein E; MDP, monomeric derivatives of paeoniflorin; PcTx1, Pslogotoxin-1; HQH, Huai Qi Huang; BHGZD, Baihu-Guizhi decoction; JWJG capsule, Jinwujiangu capsule.

### 4.1 Drug targeting strategies

The NLRP3 inflammasome is closely associated with various arthritic conditions, and its abnormal activation is of great significance for the inflammatory responses as well as the development of immune diseases, and is a therapeutic target for the alleviation of inflammatory bone diseases (8). Among the drug treatment strategies for arthritis, drugs that directly block and indirectly affect the NLRP3-mediated pyroptosis pathway can be classified according to their different activation types. First, NLRP3 inflammasome direct inhibitors include: 1) NLRP3 direct inhibitors, such as icariin (ICA), CY-09, MCC950, and leojaponin; 2) caspase direct inhibitors, such as Ac-YVAD-cmk and NSC697923.

Various molecular mechanisms have been implicated in the NLRP3 activation pathway, including potassium efflux, calcium signaling, lysosomal damage, and mitochondrial dysfunction (17). Thus, depending on their mechanism of action, indirect inhibitors of the NLRP3-mediated pyroptosis pathway can be classified as:1) NF-κB inhibitors, such as punicalagin, morroniside, loganin, dietary fatty acid, Lico A and baicalein E (BF-2); 2) ROS or mitochondrial function inhibitors, such as monomeric derivatives of paeoniflorin (MDP), A3AR agonist (CF101), gallic acid, corilagin, resveratrol (Res); 3) ASIC1a inhibitors, such as amiloride and Pslogotoxin-1 (PcTx1); 4) P2Y14 receptor antagonists, such as HQL6 and 3-amide-5-aryl benzoic acids; 5) GSDMD inhibitors, such as necrosulfonamide (NSA); 6) PKA activators, such as wedelolactone; 7) AMPK activators, such as SDF-1. In addition, a number of studies have reported that Chinese herbal ingredients can interfere with the NLRP3 inflammasome in the treatment of RA, including Huai Qi Huang Granules (HQH), Baihu-Guizhi decoction(BHGZD), Jinwujiangu Capsule (JWJG capsule). Traditional Chinese medicine ingredients can affect the NLRP3 inflammasome to activate and reverse the imbalance of the inflammatory immune system during the onset and progression of RA, but its specific mechanism is still unclear.

### 4.2 miRNAs targeting strategies

miRNAs are noncoding small RNA molecules composed of 18-23 nucleotides that can negatively regulate gene expression (71) and are widely involved in inflammation and pyroptosis pathway. And a number of studies have reported that miRNAs play an important role in the NLRP3-mediated pyroptosis pathway. Different miRNAs are known to play regulatory roles in the progression of OA (miR-107, miR-155, miR-140-5p, miR-326, miR-219a-5p), RA (MiR-144-3p, MiR-135b-5p) and GA (miR-223-3p). Therefore, we summarize recent studies related to miRNAs/NLRP3 signaling with function of regulating the development of arthritis (**Table 3**).

**Table 3:** Targeting miRNAs to regulate NLRP3-mediated pyroptosis in arthritis.

Types of miRNAs	Target gene	Possible upstream or downstream pathways	Related arthritis	Ref
miR-144-3p	PTEN	PTEN/PINK1/Parkin/NLRP3/Caspase-1	RA	(44)
miR-135b5p	SIRT1	SIRT1/NLRP3	RA	(72)
miR-155	SMAD2	SMAD2/NLRP3/Caspase-1	OA	(28)
miR-140-5p	CTSB	CTSB/NLRP3	OA	(73)
miR-326	HAC3 and STAT1	miR-326/HAC3 and STAT1/NF-κB/NLRP3	OA	(74)
miR-219a-5p	FBXO3	FBXO3/NLRP3	OA	(75)
miR-223-3p	NLRP3	miR-223-3p/NLRP3	GA	(71)
miR-107	Caspase-1	miR-107/Caspase-1	OA	(76)

### 4.3 New targeting strategies

Various types of arthritis exhibit increased joint inflammation and release of inflammatory cytokines, and understanding the mechanisms of pro-inflammatory cytokine expression and exploring potential therapeutic approaches offers new strategies for the management of these diseases. Complement activation contributes to inflammation, which aids in the pathogenic processes of autoimmune disorders. PTX3 regulates complement function by interacting with C1q and factor H, playing a crucial role in the innate immune system (61). Wu et al. (61) highlight the role of complement C1q and its ligand PTX3 in enhancing the synthesis of inflammatory cytokines, specifically IL-6. This leads to the over-activation of NLRP3 and pyroptosis induced by PTX3 and C1q, creating a positive feedback loop. Their findings suggest a new potential treatment strategy targeting pyroptosis-mediated persistent inflammatory cytokine release.

GSDMD-targeting treatment with siRNA is one of the current research hotspots for blocking the burst release of inflammatory cytokines in the NLRP3-mediated pyroptosis. PEI (polyethylenimine) combined with cholesterol (Chol) as the hydrophobic component has been discovered to be an excellent carrier for gene delivery due to its high transfection efficiency and low cytotoxicity. Ye et al. (64) assembled synthetic PEI-Chol (cholesterol grafted polyethylenimine) with GSDMD small interfering RNA (siRNA) to form PEI-Chol/siGSDMD polyplexes, and demonstrated the role of siGSDMD-loaded PEI-Chol in MSU-induced pyroptosis and acute inflammatory cascade, offering a potential therapeutic strategy for molecular treatment of acute GA.

DNA polymerase β (Polβ) has an important role in autoimmunity, and DNA damage caused by Polβ deficiency can induce RA macrophage pyroptosis, thereby aggravating the severity of the disease (58). Gu et al. (58) discovered that Pol β controls RA by suppressing macrophage pyroptosis through the cGAS/STING/NF-κB pathway. This finding strongly supports the significant involvement of Pol β in the advancement of RA, indicating its potential as a viable target for preventing and treating RA and related autoimmune disorders.

Zinc finger protein A20 plays a role in promoting cell proliferation and inflammatory response, and is involved in the regulation of the body’s innate and adaptive immunity. The A20 gene is strongly associated with the development of numerous inflammatory diseases. Walle et al. (43) discovered that A20 hinders the activation of NLRP3 inflammasome by decreasing the levels of NLRP3 expression. Consequently, the etiology of arthritis in A20myel-KO mice is attributed to the excessive activation of NLRP3 inflammasome. A20 is a novel negative regulator of NLRP3 inflammasome activation, and targeting A20 expression may provide a new strategy for the treatment of inflammatory bone diseases such as rheumatoid arthritis.

## 5 Conclusions and perspectives

The NLRP3 inflammasome is a major sensor of non-infectious inflammatory diseases and is an important initiator of acute and chronic inflammation. Inflammatory cytokines such as IL-1β and IL-18 released in the NLRP3-mediated pyroptosis are secreted into extracellular tissues and participate in the subsequent inflammatory cascade response (77). A series of studies have reported the important role of the NLRP3 inflammasome in the pathogenesis of bone and joint diseases. Its activation is inhibited to reduce the inflammatory response of the osteoarthritis and to improve the local condition of the joint, demonstrating the feasibility of inhibiting the activation of NLRP3 inflammasome and the release of associated cytokines as a new target for the treatment of arthritic disorders. Although the NLRP3 inflammasome has shown potential application value in the treatment of arthritic disorders at this stage, it still needs to be further clarified. First, a number of studies have reported the potential value of the NLRP3 inflammasome in arthritis, but the mechanism involved has not been fully elucidated. The possible synergy between NLRP3-mediated pyroptosis and unknown cell signaling involved in the regulation of arthritis development remains a critical challenge for the development of NLRP3 inflammasome-based therapies. The mode and mechanism of NLRP3 inflammasome activation requires further support from high quality studies. Second, the current researches on the role of the NLRP3 inflammasome in bone and joint diseases are mostly derived from cell and animal experiments, and clinical researches are relatively scarce. The inability of cellular or animal models to simulate the microenvironment within humans may result in these studies lacking real conviction. Notably, long-term sustained inhibition of NLRP3 inflammasome may impair normal immune function in the body, which may ultimately produce irreversible damage.

In this review, we explored the biological properties of the NLRP3 inflammasome and the role and research progress of the NLRP3-mediated pyroptosis in sterile arthritis. We described approaches to modulate NLRP3 inflammasome for the treatment of sterile arthritis, including drugs, miRNAs and novel therapeutic strategies. Overall, researches on the mechanism of the NLRP3 inflammasome and arthritis is still in its infancy. In-depth study of the role of NLRP3 inflammasome in arthritis may provide guidance on therapeutic approaches and potential targets for arthritis. This provides more strategies for the diagnosis and treatment of arthritis in clinical practice in the future.

## Author contributions

YX: Conceptualization, Writing – original draft, Writing – review & editing. LZ: Funding acquisition, Supervision, Validation, Writing – original draft, Writing – review & editing.
